# Response to Letter to the Editor: “Comment on “Serum Hepcidin and Soluble Transferrin Receptor in the Assessment of Iron Metabolism in Children on a Vegetarian Diet””

**DOI:** 10.1007/s12011-018-1482-z

**Published:** 2018-09-03

**Authors:** Jadwiga Ambroszkiewicz, Witold Klemarczyk, Joanna Mazur, Joanna Gajewska, Grażyna Rowicka, Małgorzata Strucińska, Magdalena Chełchowska

**Affiliations:** 1 Screening Department, Institute of Mother and Child, Kasprzaka 17A, 01-211 Warsaw, Poland; 2 Department of Nutrition, Institute of Mother and Child, Kasprzaka 17A, 01-211 Warsaw, Poland; 3 Department of Child and Adolescent Health, Institute of Mother and Child, Kasprzaka 17A, 01-211 Warsaw, Poland

Dear Editor,

We read the comments on our article “Serum hepcidin and soluble transferrin receptor in the assessment of iron metabolism in children on a vegetarian diet” written by Piotr Rzymski and Tomas Ganz with a great interest. First of all, we thank them very much for excellently reviewing our paper and presenting an interesting alternative point of view.

In our paper, we suggested that inclusion of novel markers such as hepcidin and soluble transferrin receptor (sTfR) can be useful in precise assessment of iron metabolism.

Due to the fact that iron deficiency in children is a global problem and that iron plays an important role in many metabolic processes, we decided to undertake this subject knowing that iron deficiency may concern both children following traditional as well as vegetarian diets. Some researchers claim that in vegetarians the intake of iron needs to be higher (up to 80%) to overcome the lower availability of iron from plant sources. Similar to Rzymski and Ganz, we also question this recommendation.

On the basis of our results, we suggested in a classical manner that decreased serum hepcidin and elevated sTfR levels might be the reason for subclinical iron deficiency in the studied prepubertal vegetarian children. This is our hypothesis, and we intend to do a follow-up on our children in a few years to ascertain whether they have normal values of hematological and biochemical parameters related to iron metabolism. This research would be particularly important due to the fact that some of the children (especially girls) will be in pubertal period when the need for iron is increased.

We thank the authors for presenting an alternative interpretation of lower hepcidin and higher sTfR which may reflect a clinically benign form of adaptation for more efficient iron absorption and utilization. It is a very interesting point of view, and we agree with the authors that this might be the case in vegetarians who consume high amounts of ascorbic acid in their diets. However, we are wondering if this compensation is not caused by subclinical iron deficiency.

We know that in case of vegetarian diets, compensatory effect may exist. This situation concerns not only iron metabolism but also bone metabolism. In another one of our studies (not published yet), we observed higher serum level of carboxylated osteocalcin in vegetarian children compared with omnivorous children. Osteocalcin is the main non-collagenous protein, which reflects osteoblast activity and bone formation and which may exist in two forms: carboxylated osteocalcin and undercarboxylated osteocalcin. Fully carboxylated osteocalcin plays an active regulatory role in bone mineralization. Since in vegetarian diet intake of vitamin K is generally higher than in traditional diet, elevated level of carboxylated form of osteocalcin might be a compensatory effect of vegetarian diet on bone health.

